# A Taxonomic Hierarchy of Blockchain Consensus Algorithms: An Evolutionary Phylogeny Approach

**DOI:** 10.3390/s23052739

**Published:** 2023-03-02

**Authors:** Heesang Kim, Dohoon Kim

**Affiliations:** Department of Computer Science, Kyonggi University, Suwon-si 16227, Republic of Korea

**Keywords:** fault tolerance, Byzantine fault, crash fault, distributed system, blockchain, consensus, classification, taxonomic hierarchy, taxonomic rank, taxonomic tree

## Abstract

Countless endeavors have been undertaken to address the Byzantine Generals Problem, a generalization of the Two Generals Problem. The emergence of proof of work (PoW) for Bitcoin has led to various consensus algorithms diverging, and comparable existing consensus algorithms are being gradually utilized interchangeably, or only developed for each specific application domain. Our approach employs an evolutionary phylogeny method to classify blockchain consensus algorithms based on their historical development and current usage. To demonstrate the relatedness and lineage of distinct algorithms, as well as to support the recapitulation theory, which posits that the evolutionary history of its mainnets is mirrored in the development of an individual consensus algorithm, we present a taxonomy. We have created a comprehensive classification of past and present consensus algorithms that serves to organize this swift consensus algorithm evolution period. By recognizing similarities, we have compiled a list of different verified consensus algorithms and performed clustering on over 38 of these. Our new taxonomic tree presents five taxonomic ranks, including the evolutionary process and decision-making method, as a technique for analyzing correlation. Through the examination of the evolution and utilization of these algorithms, we have developed a systematic and hierarchical taxonomy that enables the grouping of consensus algorithms into distinct categories. The proposed method classifies various consensus algorithms according to taxonomic ranks and aims to reveal the direction of research on the application of blockchain consensus algorithms for each domain.

## 1. Introduction

Blockchain technology and decentralized systems are intimately connected, as blockchains represent one of many variants of decentralized systems. Decentralized systems operate on a network of nodes, with each node carrying equal responsibility and authority. They operate without central control or decision making, with nodes communicating directly to validate transactions and achieve consensus. Blockchains utilize consensus algorithms and cryptography to maintain a secure and transparent ledger of transactions. Transactions are verified and recorded by multiple nodes in a blockchain, making it virtually impossible to tamper with once added to the chain. As a result, blockchains are a reliable and tamper-proof platform for the exchange and storage of data, assets, and other forms of value. The relationship between decentralized systems and blockchain highlights the potential of decentralized systems to provide secure, transparent, and reliable solutions for various applications. By leveraging the strengths of decentralized systems and blockchain technology, it is possible to develop systems that are less vulnerable to manipulation, censorship, and single points of failure, providing greater privacy and security for users.

Consensus algorithms play a critical role in verifying and adding transactions and blocks to the blockchain, ensuring the system remains tamper-proof and secure. While different consensus algorithms employ different approaches to verify transactions and blocks, their goal is to provide a secure, reliable, and efficient way for multiple participants to reach consensus in a decentralized system. The concept of distributed systems dates back to the early days of computing when scientists were exploring systems that could function despite the failure of some of their components. The earliest distributed systems were developed in the 1960s and 1970s, primarily for use in scientific and academic settings. With the advent of the internet and client–server computing in the late 1980s and early 1990s, distributed systems became more widely used. As networked computers became more readily available, it became feasible to create systems that could operate on multiple machines and share resources over a network.

Blockchain technology, on the other hand, emerged as a result of the creation of cryptocurrencies, with Bitcoin [1] being the most well-known example. The proof of work (PoW) consensus algorithm, created by Markus Jakobsson and Ari Juels [2], was among the first forms of consensus algorithms and was employed to address the problem of double-spending in the early stages of cryptocurrency. A PoW algorithm mandates that participants must perform a certain amount of computational work to validate transactions and add blocks to the blockchain, ensuring the network remains secure, decentralized, and resilient to malicious attacks. Satoshi Nakamoto introduced Bitcoin [1] in 2009, which was designed to be a decentralized digital currency that allowed value transfer from one person to another without intermediaries. To achieve this, Satoshi Nakamoto combined concepts from cryptography, game theory, and distributed systems to create the first blockchain, which maintained a secure and transparent ledger of all Bitcoin transactions. Since the inception of Bitcoin, the blockchain concept has been applied in various domains, including smart contracts, supply chain management, and voting systems. Additionally, the technology has catalyzed the development of novel types of decentralized systems and networks, such as decentralized autonomous organizations (DAOs) and decentralized finance (DeFi) systems. Today, blockchain technology is one of the most crucial and innovative technologies of the 21st century, revolutionizing the way we store, track, and exchange data, assets, and other forms of value.

Consensus algorithms used to achieve fault tolerance [3,4,5,6,7,8] in distributed systems, including blockchains, occur according to algorithm-based, predefined state transition rules. In a blockchain-based system, the shared state is the blockchain, and the state transition rules are the consensus algorithm, which is the blockchain protocol. The most important problem, a Byzantine fault [9,10], occurs due to software bugs or because of Byzantine nodes being included in the network and communicating. The problems of being able to lie, provide ambiguous responses, or completely mislead the messages of other nodes involved in the consensus protocol must be addressed so that consensus algorithms work correctly and reach consensus within a distributed system. Blockchain consensus algorithms are continuously being researched in an effort to make rational and efficient decisions. However, there is no standard for retroactively classifying the newly researched and proposed blockchain consensus algorithms. There is no classification standard for rules agreed upon in a decentralized environment. Due to the diversity of blockchain consensus algorithms, it is difficult to determine the appropriateness of each blockchain technology application domain, and it is also difficult to identify the characteristics and limitations of each blockchain consensus algorithm. The Two Generals Problem [11] is a difficult problem in fault tolerance in the research fields of past distributed systems. Currently, various consensus algorithms are attempting to efficiently solve the Byzantine Generals Problem [9,10], where the Two Generals Problem [11] has become more common. Currently, various consensus algorithms have diverged from the Byzantine fault tolerance (BFT) [9,10,12] method, which was a consensus algorithm that diverged through continuous research on an efficient fault tolerance method until PoW was applied to Satoshi Nakamoto’s Bitcoin [1] in 2009. Currently, they are similar to one another, and research is being conducted to derive the best consensus algorithm by mixing them in a hybrid form. However, as the blockchain trilemma emerges, the existing consensus algorithm has gradually been mixed and used, because there is no perfect consensus method applicable to any blockchain yet. Rather than continuing research by mixing indiscriminate consensus algorithms, systematic consensus algorithm research through consensus algorithm classification will accelerate the development of the fault tolerance field in the future. In fault tolerance [3,4,5,6,7,8,13,14,15], a field of distributed systems, blockchain refers to a method of sharing computer resources or contents that are connected horizontally rather than using the existing server–client method, which is a relatively vertical connection. Since the server–client method has a single point of failure (SPOF) structure, the service is unavailable in the event of a server failure. However, blockchain guarantees high availability or fault tolerance with little or no disruption to the service even if one of the countless nodes, which are connected to computers, fails. Blockchain is a kind of distributed system, but it has a serious problem in that the participation of malicious peers breaks mutual trust and completely disintegrates the system. Reliability adds to the integrity of the system, and when trust is broken, the continuity of the peer-to-peer (P2P) system collapses. With the advent of blockchain, reference is made to a distributed ledger and P2P system that utilizes software elements composed of algorithms that are agreed upon using cryptographic techniques and security technologies to secure and maintain the integrity of the system.

The classification and categorization of various types of blockchain systems, their components, and attributes through a blockchain taxonomy is necessary to comprehend the distinct characteristics and capabilities of each blockchain type. This, in turn, helps in decision making for selecting the most suitable blockchain for specific use cases, thereby reducing confusion. Moreover, blockchain taxonomy allows for easy comparison and the evaluation of various blockchain systems, and aids in the development of industry standards and best practices. However, due to the diverse perspectives and priorities of different communities and stakeholders, there is currently no clear consensus on a comprehensive taxonomy for blockchain technology. Blockchain is a rapidly evolving field with different definitions, applications, and uses, making it challenging to develop a universal taxonomy that covers all aspects of the technology. As blockchain matures and becomes more widely adopted, it is expected that a consensus will be reached, and a taxonomy will be developed to categorize and organize the different types of blockchain systems.

In the classification of the blockchain consensus algorithms proposed in this study, there are three significant limitations and problems:

1. The lack of clarity in the classification system of blockchain consensus algorithms makes it impossible to categorize newly researched blockchain consensus algorithms. This uncertainty in the taxonomy of consensus algorithms also makes it challenging to determine the characteristics of current and future consensus algorithms, even when examining the relevant documents.
2. Researchers of new consensus algorithms find it difficult to trace the latest research in the fault tolerance field, and thus cannot identify the genealogy for future research. To trace the root of blockchain research, it is necessary to examine the Byzantine Generals Problem, which is one of the challenges in the field of fault tolerance. Byzantine fault tolerance (BFT) [9,10,12] was proposed as a solution to the Byzantine Generals Problem and was subsequently adapted for the blockchain field.
3. State-of-the-art consensus algorithm research largely focuses on improving performance and shares similarities with previous research purposes, making it challenging to discern the fundamentals of each consensus algorithm for research and development and the contribution of technology applications. Since the proposal of the PoW consensus algorithm, countless unclassified consensus algorithms have gradually been mixed, making it challenging to understand research progress. As a result, it is difficult to identify where to start research and what references to use when proposing an ideal blockchain consensus algorithm, even if a new consensus algorithm is proposed.

In summary, our paper provides a significant contribution to the field of blockchain, through the introduction of an innovative and comprehensive taxonomy and classification method for blockchain consensus algorithms based on an evolutionary phylogeny approach.

As a means of resolving the limitations and issues associated with current blockchain consensus algorithms, this study seeks to identify serial correlations in the results of research and development of such algorithms [3,4,5,6,7,8,13,14,15] and to examine trends or seasonality among different consensus algorithms. We have examined and refined consensus algorithms [1,2,16,17,18,19,20,21,22,23,24,25,26,27,28,29,30,31,32,33,34,35,36,37,38,39,40,41,42,43,44,45,46,47,48] and 41 mainnets [1,18,25,30,31,32,36,37,38,39,40,49,50,51,52,53,54,55,56,57,58,59,60,61,62,63,64,65,66,67,68,69,70,71,72,73,74,75,76,77], referring to various published papers and whitepapers on websites. We aimed to perform a comprehensive review of the literature without limiting the references, which was challenging to complete on prior articles. Most of the references in the blockchain domain are websites due to the relative novelty and evolution of the field. Websites are more accessible and offer real-time information, making them a convenient source of knowledge for those interested in blockchain technology. Additionally, since blockchain technology is still in its early stages and is continuously evolving, websites are capable of providing state-of-the-art information that may not be accessible in conventional research papers or articles. However, as the technology matures, there is likely to be an increasing number of research papers and articles, offering a more in-depth understanding of blockchain technology. Furthermore, this study aims to summarize and present the taxonomy of the blockchain consensus algorithm based on five taxonomic ranks. By presenting the current state of research on blockchain consensus algorithms and the level of algorithm research, we can identify the limitations of existing consensus algorithms and any additional points. Our blockchain taxonomic method is presented in five steps:

1. Conducting a comprehensive review of the existing literature: Our survey consists of existing taxonomies, definitions, and classifications of blockchain to comprehend the current state of the field and identify areas for improvement.
2. Developing a set of criteria and categories: Based on our review, we develop a set of criteria and ranks that can classify different types of blockchain systems, ensuring that they are comprehensive, objective, and relevant to the scope and objectives of the taxonomy.
3. Applying the ranks to representative blockchain systems: We evaluate the classification of human decision-making methods and forms of government criteria and categories by applying them to a representative sample of blockchain systems to see how well they perform in classifying the systems.
4. Presenting the taxonomy and its results: We discuss the results of the classification, highlighting any interesting patterns or insights that emerged from the analysis.
5. Maintaining and updating the taxonomy in future works: We plan to review and update the taxonomy regularly as the field evolves and new blockchain systems emerge.

The rest of this study is structured as follows: Section 2 summarizes the relevant background and progress made over the past two decades, providing a brief introduction to the domain of distributed systems, fault tolerance, and the rise of blockchain in Section 2.1. Section 2.2 describes the consensus algorithms used in the last decade, focusing on documented technologies and research. In Section 2.3, we classify blockchain consensus algorithms to date, and describe existing studies on the challenges of each technology application area or algorithm. Finally, in Section 3, we present our attempt to resolve the fault tolerance domain on the timeline based on the current research trends in Section 2, presenting taxonomical ranks and a taxonomy based on the proposed classification system, offering a method to classify all consensus algorithms. This study is concluded with overall suggestions for future research in Section 4 and Section 5.

## 2. Related Work

### 2.1. Field of Fault Tolerance and Blockchain

The use of research in the field of fault tolerance in distributed systems [3,4,5,6,7,8,13,14,15] has allowed for the historical tracking of the blockchain consensus algorithm from the past to the present. Failure models in distributed systems [3,4,5,6,7,8], which are traditionally classified into six types, also have a hierarchical structure [78,79], where larger classes encompass smaller ones. The distributed system establishes a target failure model and discontinues processing when a failure corresponding to the upper layer occurs. Table A1 of Appendix A outlines the six faults and their design faults. Among the six levels of faults, this study exclusively focuses on the authentication-detectable Byzantine fault and the scope of the blockchain consensus algorithm, which is an effort to resolve the Byzantine fault. Table 1 juxtaposes the Byzantine fault failure with the Byzantine Generals Problem noted by Lamport [9,10] and the previous fault failure. In a synchronous network [80,81,82], the information propagation time of the network is fully controlled in the process of transmitting and propagating the message, and all nodes modify or anticipate the arrival time of the messages they have sent. Any delay that impedes the arrival time must be predicted accurately and cannot occur. Distributed systems alter states by exchanging messages between various nodes, where it cannot be assumed that all nodes are normal. An asynchronous network [83,84,85,86] refers to a scenario in which the information propagation time of the network cannot be predicted during message transmission and propagation. It is hypothesized that messages between nodes may be entirely lost or disappear due to the presence of many botnets and denial of service (DoS) attacks on the network. Although distributed systems do not have the problem of SPOF, the likelihood of issues arising is higher than that of systems managed by a single node, since multiple nodes manage system resources in a distributed manner, making it impossible to handle all errors in reality. In general, distributed systems are constructed to define errors they can tolerate, but they cannot guarantee operation when other errors occur. The type of error that is tolerated at a given time is referred to as the failure model of the system. To address this issue, it is necessary to examine the failure model presented in the studies by Denning [14] and Linden [15] in the field of fault tolerance in distributed systems.

The foremost disparity between the two classifications depicted in Table 1 lies in their consideration of message content alteration. Faults akin to omission, such as message non-arrival, prevail until the fail-stop, crash, omission, and performance fault levels. However, authentication-detectable Byzantine and Byzantine faults, which include the Byzantine Generals Problem [9,10], presume the arrival of a message but the transmission of its content in a tampered state. Of these, this research explores only three models that underpin contemporary consensus algorithms. The crash failure model assumes that a node that fails to respond to a request always crashes. When a node is unresponsive, the problem can be resolved by replacing the failed node with a new one. Nevertheless, crash failure is unrealistic in Internet-scale distributed systems since messages may fail to arrive for reasons other than node crashes. Therefore, crash failure is typically used in multi-core processor inter-processor communication or distributed systems within a data center.

The fail-stop failure model, crash failure model, omission failure model, and performance failure model are all trusted once a message arrives. However, certain nodes may have bugs or hardware problems that result in messages being partially transmitted or modified due to man-in-the-middle attacks. Byzantine failures arise when nodes inconsistently send incorrect messages, making it challenging to solve the problem, even when some messages are transmitted correctly. The Byzantine Generals Problem is one of the most difficult challenges in the fault tolerance field. BFT [9,10,12] is a distributed computing theory that has been developed to achieve consensus even if a Byzantine node is present within the network. Achieving perfect consensus in an asynchronous network is a typical challenge in distributed computing. Because it is challenging to handle all Byzantine failures, the authentication-detectable Byzantine failure model, which addresses only verifiable Byzantine failures among Byzantine failures, is the first model that is studied. The authentication-detectable Byzantine failure model resolves the problem by implementing an error detection scheme or using asymmetric encryption. Most internet-scale distributed systems currently in use assume an authentication-detectable Byzantine failure model.

However, blockchain networks adopt a Byzantine failure model that considers all Byzantine failures [87,88], seeking to address the most intractable problem assumed in conventional distributed systems, the Fischer, Lynch, and Paterson (FLP) impossibility [89]. The FLP impossibility problem was circumvented by implementing a blockchain structure and introducing an algorithm known as proof of work, making Bitcoin [1] the first realized solution to the Byzantine Generals Problem. Safety is a characteristic that two nodes without faults should not agree on different values, and nodes without issues should not end up in an infinite loop, while the state change must be completed. Liveness is the property that all nodes achieve consensus without difficulty. Fischer, Lynch, and Paterson demonstrated that in an asynchronous network environment, there was no consensus algorithm that satisfied both the safety and liveness requirements if even one node could fail. Communication delays in asynchronous networks are unpredictable, making it impossible to determine whether a node has failed due to a specific issue or whether the response time is due to network delays. Most existing distributed systems deliberately ignore Byzantine failures, and most nodes do not consider abnormal behavior, malicious behavior, or hacking. Several proposals and projects attempted to create a token economy in a decentralized environment prior to Bitcoin, but none were successful. Efficient blockchain consensus algorithms, such as BFT-based consensus algorithms and practical Byzantine fault tolerance (PBFT) [90,91], are currently being investigated.

### 2.2. Scope

The present study focuses on a survey of consensus algorithms, categorized into three types of failure models, including authentication-detectable Byzantine faults, Byzantine faults, and crash faults. Its objective is to review the evolution of consensus algorithms employed in blockchain, ranging from the Nakamoto consensus, which prioritizes liveness over safety, to the Tendermint [16,17] consensus, which emphasizes safety over liveness. The purpose of this study is to classify consensus algorithm systems and facilitate their selection by researchers, depending on the level of safety and liveness required for the type of service being implemented. The proposed taxonomy is expected to assist in designing fault tolerance against treachery, where a specific node intentionally withholds message transmission or transmits false data in an asynchronous network in the future.

#### 2.2.1. Authentication-Detectable Byzantine Fault Tolerance

The consensus algorithm that is designed with consideration of authentication-detectable Byzantine fault tolerance guarantees absolute finality. In the second approach to bypassing the FLP impossibility [89], known as the method of safety over liveness, the BFT-based consensus algorithm and PBFT-based protocol consider a transaction to be completed immediately when a block is included and added to the blockchain, with the block only being committed when the leader proposes it and a sufficient percentage of the validator committee approves it. As for the various consensus algorithms currently in use, apart from error detection schemes in Internet-connection-based distributed systems, the problem of achieving consensus in a malicious failure scenario was introduced by Lamport, Shostak, and Pease in a classic 1982 study [9]. This problem is at the heart of authentication-detectable Byzantine failure, and Lamport presented the Byzantine Generals Problem in his study to initiate research to solve the Byzantine fault. A method similar to verifying messages and sharing contents only by trusted nodes was also proposed. An evolution of this approach is Paxos [92,93,94,95], which is a group of protocols for solving consensus problems in networks of untrusted processors. The consensus protocol, validated by Fred Schneider [96], is based on the state-machine approach to distributed computing, proposed by Leslie Lamport. The state-machine approach is a technique for transforming an algorithm to be implemented in a fault-tolerant distributed system, and it led to the development of the PBFT [90,91] consensus algorithm, which was proposed in 1999 as a form of BFT [9,10,12], improved in speed and practicality. The consensus is verified through a two-step process, and the reliability of the consensus is mathematically guaranteed when Byzantine nodes make up less than 33% of the total. This method is a proof method developed so that all nodes achieve a successful consensus in an asynchronous system where Byzantine nodes, which intentionally deliver incorrect information without performing the promised actions in the blockchain system, may exist. Currently, the PBFT [90,91] model is being applied in various permissioned and permissionless blockchain systems to ensure fault tolerance between nodes in a distributed environment.

#### 2.2.2. Byzantine Fault Tolerance

In order to achieve a comprehensive classification, it is essential to investigate consensus algorithms developed on the basis of Byzantine fault tolerance (BFT) [9,10,12]. BFT [9,10,12] is a consensus algorithm that enables decentralized networks to reach an agreement even when some of its nodes are malicious or faulty. During the voting process in BFT, each node has a single vote, and the majority of nodes must agree on a single value. Conversely, authentication-detectable BFT is a variant of BFT that enhances security by requiring nodes to authenticate each other before participating in the consensus process. This extra step helps prevent malicious nodes from disrupting the consensus process by ensuring that only legitimate nodes can participate in the voting process. The adversary tolerance of a BFT algorithm determines the number of nodes that can behave maliciously or fail and still ensure a correct consensus. If a BFT algorithm can tolerate up to 33% of the nodes being faulty, it is said to have a 33% adversary tolerance. The adversary tolerance of an authentication-detectable BFT system depends on the authentication mechanism and the BFT algorithm used. However, such systems are expected to have a higher adversary tolerance compared to regular BFT systems, as they add an extra layer of security by requiring node authentication. By preventing malicious or faulty nodes from participating in the consensus process, authentication-detectable BFT systems can better resist attacks and prevent compromised consensus processes. Therefore, the adversary tolerance of authentication-detectable BFT systems may be higher than that of regular BFT systems. Table 2 summarizes the differences between authentication-detectable BFT and BFT.

Probabilistic finality is a method that has been suggested to address the limitations of Byzantine fault tolerance (BFT) [9,10,12], whereby a certain amount of time is required before the recording is established and the transaction becomes finalized and irreversible. BFT is characterized by the safety-over-liveliness feature, meaning that it will not produce a block if there is a possibility of an incorrect consensus. The basic BFT [9,10,12] method does not guarantee liveness since a block cannot be created without reaching a consensus in an asynchronous network. PoW [1,2], a consensus algorithm that was later developed in BFT [9,10,12], is the consensus algorithm used in Bitcoin and is also known as the Nakamoto Consensus. According to the PoW method, a block is produced whenever a miner discovers a nonce to a difficult problem. The longest chain thus formed is considered a valid and canonical chain. The PoW method provides liveness over safety among the methods of bypassing FLP impossibility [89], and although an incorrect agreement can occur, an agreement is reached nonetheless. All nodes verify and approve the found hash value and save the transaction details in the block, making it difficult to fake the transaction details because they must be approved by all nodes. The PoW [1,2] consensus algorithm captures the essence of blockchain decentralization the best.

Proof of stake (PoS) [18] is another consensus algorithm that provides decision-making authority proportional to the percentage of the stake held in the cryptocurrency. In PoS [18], nodes holding more coins have more opportunities to participate in block creation, and the reward for block creation is proportional to the number of coins held. Unlike the PoW method, the PoS [18] method does not require approval from all nodes, allowing for faster transaction processing speed and reduced power consumption. Delegated proof of stake (DPoS) [19], on the other hand, is a consensus algorithm in which cryptocurrency holders exercise their voting rights in proportion to their stakes to choose their representatives and make decisions based on consensus among these representatives. In the DPoS [19] method, only a few representative nodes need to approve transaction details, resulting in much faster processing speeds. However, the DPoS [19] method has the drawback of being dominated by a few representative nodes in the blockchain ecosystem if the voting rate of general nodes is low.

#### 2.2.3. Crash Fault Tolerance

Crash fault tolerance (CFT) is a fault-tolerance technique that guarantees the ability to achieve consensus even if certain components fail. This allows the algorithm to continue the process and reach consensus in the event of component failure. CFT is particularly advantageous for single companies. Presently, Hyperledger Fabric [20,97,98,99,100,101,102,103] employs CFT, and organizational unit blockchain network implementations also utilize Kafka [104].

### 2.3. Previous Blockchain Taxonomic Research

The aforementioned solutions share a common attribute, which is the implementation of a well-designed consensus algorithm to ensure fault tolerance against node failures, network splits, message delays, messages arriving out of order, and corrupted messages. This has led to the proposal of various algorithms, each of which comprises different elements such as synchronization requirements, message broadcasting, error handling, malicious nodes, performance, and the security of exchanged messages. In a blockchain network, achieving consensus is crucial as it ensures all nodes in the network agree on a consistent global blockchain state. Consensus protocols are evaluated based on their safety, activity, and fault tolerance, which determine their applicability and efficacy. Safety is established when all nodes produce the same output that is valid according to the protocol rules. Activity is ensured when all non-faulty nodes eventually produce a value. Fault tolerance is provided when consensus protocols recover from the failure of participating nodes.

Previous blockchain taxonomic research aimed to efficiently classify and select consensus algorithms, leading to the review of previous blockchain taxonomic studies in this research. The well-organized taxonomic research was analyzed to identify the commonalities between each study and determine the direction that blockchain consensus classification research should take. Table 3 provides a summary of the main blockchain taxonomy since 2009, when the existing classification method was first identified.

The blockchain field has seen many surveys and taxonomies [104,105,106,107,108,109,110,111,112,113,114,115,116,117,118] concerning consensus algorithms. However, recent studies addressing the correct classification of these algorithms are scarce. The number of fixed classification criteria made it challenging to distinguish the characteristics and ancestral relationships of each algorithm, which hampers future research, making it similar to previous studies.

Group 1 in Table 4 covered attempts to classify consensus algorithms and set categories for them [105,106,108,110,113,114]. The authors proposed classifying using proof-of-something (Proof-of-X; PoX), which could classify algorithms such as proof of work (PoW) [1,2], proof of stake (PoS) [18], and delegated proof of stake (DPoS) [19] that emerged before the early 2010s. However, it was difficult to infer the connection between practical Byzantine fault tolerance (PBFT) [90,91] and Byzantine fault tolerance (BFT) [9,10,12]. Group 2 taxonomic research in Table 4 focused on creating a general taxonomy, including blockchain analysis tools [107,118], applications [107,109,110,116,117,118], challenges, and threats [107,109,111,112,115,116,117,118]. Recent research [116] focused on the basics of blockchain, consensus algorithm progress, characteristics, and future development trends, but it did not present a taxonomic tree classifying consensus algorithms. Another recent study [117] presented a survey of blockchain use in IoT systems, proposed a taxonomy based on significant factors, examined widely used blockchain platforms, explored potential applications, recent advances, challenges, and future research directions. Although related studies were helpful, the taxonomy presented in this study was limited to IoT, and the taxonomic classification criteria was only based on whether the chain is public or private, leaving out other algorithms. Another article [118] discussed the potential of emerging technologies, especially blockchain and IoT, and proposed an architecture for integrating them using the latest tools and methods. It explored their principles, consensus methods, challenges, applications, and future trends. We consulted related studies but focused on the development process of blockchain consensus algorithms from the past to the present, and the objectification and classification of new consensus algorithms in the future, instead of performance analysis [106,107,109] or security challenges [108,111].

Though various attempts and methods have been proposed to classify consensus algorithms accurately, the latest consensus algorithm research direction and achievements remain unclear. The thorough review and summary of existing fault tolerance research is vital for the future of this field, especially for researchers entering it.

Our survey constitutes a departure from formal research, as the absence of a standard for the classification of consensus algorithms in a decentralized and distributed environment implies that newly developed blockchain consensus algorithms cannot be categorized conventionally. Thus, it becomes essential to familiarize oneself with the developmental status of several consensus algorithms that are currently in progress. In order to achieve the full classification of all existing algorithms, we proposed the introduction of taxonomical ranks. While consensus algorithms are designed to solve the problem of failures, the main point of the blockchain field’s most progressive attempt to address the Byzantine Generals Problem among distributed networking or distributed systems cannot be fully captured by the current classification method.

In light of the distributed system challenge that initiated Bitcoin’s [1] history, each algorithm provides a different mechanism to ensure safety, liveness, and finality. In particular, the blockchain consensus algorithm ensures safety by avoiding erroneous agreements between nodes and guaranteeing liveness through node consensus. The most effective way to solve the Byzantine Generals Problem at present is by summarizing the FLP impossibility [89] bypass methods as either liveness over safety, a redefinition of consensus, or safety over liveness, abandoning asynchrony.

Probabilistic or absolute finality are the two methods employed to ensure the finality of the blockchain. The former guarantees finality in Bitcoin by using a PoW algorithm, while the latter is utilized in BFT-based consensus algorithms. As such, a survey encompassing almost all authentication-detectable Byzantine faults and Byzantine fault consensus algorithms is necessary.

## 3. Taxonomy of Blockchain Consensus

### 3.1. Chronological Order of Blockchain Consensus

Our taxonomy is based on an evolutionary phylogeny approach, which provides a systematic and scientific way to organize blockchain consensus algorithms. Our approach enables researchers and practitioners to better understand the historical development and current state of blockchain consensus algorithms, and to identify potential directions for future research and development.

**Figure 1:**
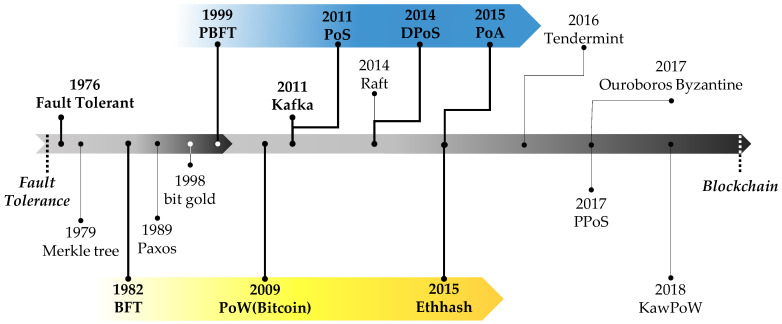
An evolutionary approach: sequential arrangement of well-known consensus algorithms.

During this study, all published consensus algorithms were thoroughly reviewed, allowing for a comprehensive overview of the release process of these algorithms over time, prior to their classification. The purpose of examining the temporal flow of the consensus algorithms prior to their complete classification was to group overlapping features among them. By investigating the release trend of the latest technology from the past, based on the mainstream, researchers were able to understand the latest technology’s big stem for each feature, and survey several variant consensus algorithms. The identification of similarities and characteristics of consensus algorithms by researchers entering this field has proven difficult. The main drawback of this is the classification of new consensus algorithms, which will continue to produce similar consensus algorithms and mixed forms. To address this issue, this study aims to completely classify various variants of consensus algorithms, including the latest ones, according to the proposed taxonomic ranks. Table 5 provides details of well-known consensus algorithms, which were selected from top-tier conference proceedings, published whitepapers, or other public documents with a minimum citation count of 150, or that include consensus algorithms that serve as important starting points for classification research. Only well-known consensus algorithms are included in Figure 1 to demonstrate the mainstream of the history of consensus algorithms.

Our approach adopts an evolutionary perspective to classify blockchain consensus algorithms based on their historical development and current usage. By analyzing the historical evolution and the current state of consensus algorithms, we establish a hierarchical taxonomy that groups them into distinct categories.

The study of fault tolerance in distributed systems has led to the exploration of the Byzantine fault tolerance solution, also referred to as state replication. This problem can be resolved by allowing a set of nodes to apply the same state transitions in the same order, with the exact order being generally unimportant as long as all nodes match. Figure 1 illustrates a genealogy of blockchain consensus algorithms, tracing back to the presentation of the fault-tolerance model [3,4,5,6,7,8] in 1976, up to the 2020s. Bitcoin [1] has its origins in the late 1970s with the development of time-stamping scheme using a hash function [122], hash trees, or Merkle trees [26,123], created by computer science to store data through linking encrypted blocks, presented and patented by Ralph Merkle. Merkle trees were utilized by Stuart Haber and W. Scott Stornetta to implement a system that would prevent the alteration of document timestamps and make them immutable [122]. This paper, published in 1991, is considered to be an important precursor to the development of blockchain technology, including Bitcoin [1]. While the paper does not specifically mention Bitcoin, the idea of using cryptographic hashes to link digital data into a chain of blocks, each containing a timestamp and a reference to the previous block, was further developed and popularized by Satoshi Nakamoto in the Bitcoin white paper of 2008. The blockchain technology underlying Bitcoin is essentially a distributed database of timestamped transactions that are secured using cryptographic techniques, and it builds upon many of the concepts introduced in Haber and Stornetta’s original paper. The Byzantine Generals Problem was introduced in a 1982 study [9] co-authored by Lamport, Shostak, and Pease, while researching fault-free distributed computer systems for satellites and airplanes, emphasizing the difficulty of operating a distributed computer system without a central control unit when there are failures and hacking attacks on some nodes. Later solutions, including Paxos [92,93,94,95,119] proposed by Leslie Lamport in 1989, focused on the replication of state when the communication channel was unreliable, and a small number of nodes exhibited failures, such as going offline permanently or sending outdated messages from the time they went offline by rebooting. Paxos protocol is similar to the protocol used for the consensus of view-stamped replication [124], first published by Oki and Liskov in 1988 in the context of distributed transactions. Practical Byzantine fault tolerance (PBFT) [90,91], introduced in a 1999 study by Miguel Castro and Barbara Liskov called “Practical Byzantine Fault Tolerance,” later influenced Tendermint [16,17] in 2018. Tendermint’s lineage was designed based on distributed computing and Byzantine fault tolerance (BFT) [9,10,12], according to a study by Ethan [16,17].

In response to the strong criticism of the centralized financial system in 2007, Satoshi Nakamoto conceived a decentralized currency system that would operate in a peer-to-peer manner without the need for a central organization, such as a bank. On 31 October 2008, Nakamoto published the Bitcoin [1] white paper and on 3 January 2009, he created the Bitcoin genesis block. Since then, the use of the blockchain technology in Bitcoin has proliferated, resulting in the creation of various cryptocurrencies. The PoW [1,2] consensus method used in Bitcoin’s launch in 2009 led to the introduction of Ethash [22] in 2015 and KaWPoW [25,49] in 2020. The PoW consensus algorithm requires network participants to solve complex mathematical problems to add new blocks to the chain. The process is energy-intensive and acts as a deterrent to prevent malicious actors from disrupting the network. The PoW algorithm helps to ensure network security and consensus on the state of the blockchain, ensuring that all participants have a consistent view of the data stored on the chain. PoW is one of the oldest consensus algorithms and was first introduced by Markus Jakobsson and Ari Juels [2]. It is used to secure transactions and create new blocks in a blockchain network by requiring nodes to perform computational work to validate transactions and add new blocks to the chain. PoW forms the basis of the original Bitcoin blockchain and is still used by many other cryptocurrencies and blockchain networks. Interest in virtual currency and blockchain technology has increased with Bitcoin, prompting the development of alternative consensus algorithms. In response to the problems of the PoW [1,2] method, discussions have emerged around the use of PoS [18] as an alternative. The concept of PoS [18] was first proposed in the Bitcointalk forum in 2011 and influenced Ouroboros Byzantine [24] in 2020. DPoS [18], proposed by Dan Larimer, was designed to establish a governance structure to facilitate joint decision making among multiple users, given that the blockchain lacks a central authority. Block generation is completed when more than two-thirds of the nodes elected as representatives agree. Compared to PoW [1,2] or PoS [18], DPoS [19] reduces the number of validators used for block generation and verification, thereby increasing speed and efficiency. Ethash [115] in 2015 utilized the Dagger–Hashimoto [125] algorithm, which was a combination of Vitalik Buterin’s Dagger algorithm [126] and Thaddeus Dryja’s Hashimoto algorithm [127]. Ethash [22] relies on the creation and analysis of large data sets called directed acyclic graphs (DAGs). Ethereum is a distributed computing platform that implements smart contract functions based on blockchain technology and is considered the most representative mainnet based on Bitcoin.

The emergence of Bitcoin in 2009 without a centralized management entity prompted a study of the blockchain consensus algorithm, revealing its high reliability in conducting trades despite unresolved issues. The subsequent temporal release and classification of consensus algorithms indicate that the ideal solution remains elusive, despite efforts to enhance scalability and build a smart contract environment during the consensus process. Notably, even PoW [1,2] has limitations that have driven blockchain consensus research in a particular direction.

### 3.2. Taxonomic Hierarchy in Blockchain Consensus Algorithms

The aim of this research is to suggest a comprehensive approach for categorizing recorded consensus algorithms [1,2,16,17,18,19,20,21,22,23,24,25,26,27,28,29,30,31,32,33,34,35,36,37,38,39,40,41,42,43,44,45,46,47,48]. Through this study, 38 consensus algorithms have been examined, and the retrospective classification of consensus algorithms has been conducted. To achieve the continuous classification of future consensus algorithms, this study recommends a total of five hierarchical levels of classification (Table 6).

**Figure 2:**
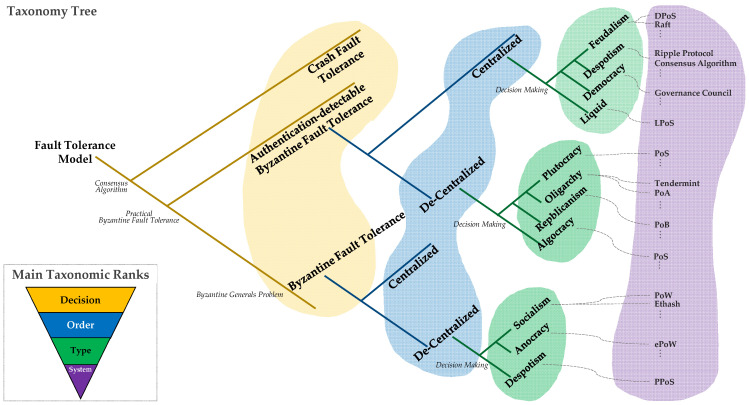
Blockchain taxonomy depicted as a hierarchical tree.

The objective of this investigation is to propose a comprehensive classification method for documented consensus algorithms [1,2,16,17,18,19,20,21,22,23,24,25,26,27,28,29,30,31,32,33,34,35,36,37,38,39,40,41,42,43,44,45,46,47,48]. Through this study, 38 consensus algorithms were analyzed, and a retrospective classification was conducted. This method is intended to be used for future consensus algorithms, ensuring that the classification process can continue. The highest classification rank in this taxonomy is the Fault rank, which is associated with the fault tolerance domain, and determines the level of fault guaranteed by each consensus algorithm. CFT, authentication-detectable BFT, and BFT [9,10,12] are the three primary classifications of consensus algorithms under this rank. The Decision rank classifies algorithms according to their common features, with decentralization as the secondary classification characteristic. The node distribution that verifies the consensus algorithm characterizes the consensus situation, with centralized decision making defined as participation by the developer of the consensus algorithm in the consensus process, and decentralized decision making as direct agreement between blockchain mainnet users. All analyzed systems are classified into centralized and decentralized. The Order rank categorizes algorithms based on the existing decision-making techniques. All blockchain consensus algorithms rely on decision-making procedures because they are designed to ensure the reliability of delivered messages among asynchronous networks. Comparing human decision-making methods to blockchain consensus algorithms revealed that there is no consensus algorithm that deviates from human decision-making methods. The Type rank is a lower level than order, and most of the consensus algorithms share similar implementations and operational methods, resulting in similar species within the consensus algorithm system. This rank classifies the mainnet in which the consensus algorithm is used, representing the most basic consensus algorithm classification system. Based on the five-step taxonomic ranks, Figure 2 presents a hierarchical taxonomy of the blockchain consensus algorithm, which begins with the Fault rank, followed by Decision rank, Order rank, Type rank, and System rank, a classification system based on the mainnet.

### 3.3. Taxonomic Hierarchy

Table 7 presents a full classification of the 38 consensus algorithms [1,2,16,17,18,19,20,21,22,23,24,25,26,27,28,29,30,31,32,33,34,35,36,37,38,39,40,41,42,43,44,45,46,47,48] and the 41 mainnets [1,18,25,30,31,32,36,37,38,39,40,49,50,51,52,53,54,55,56,57,58,59,60,61,62,63,64,65,66,67,68,69,70,71,72,73,74,75,76,77] analyzed. The consensus algorithms, spanning from the past to the present, and the mainnets built upon them are categorized according to the five taxonomic ranks. In blockchain consensus classification, taxonomic rank represents the relative level of a group of algorithms in an ancestral or hereditary hierarchy. The Kafka [20] algorithm has been included in the classification, as Kafka Streams rely on the fault-tolerant features integrated natively within Kafka [20], and have only been classified as crash fault tolerance (CFT). Since Kafka [20] consumer client is used by Kafka Streams for operations and error handling, the Streams are designed for real-time, high-volume log processing, and bypass the CFT issue by relying on at least four nodes for the Kafka-based ordering service, and BFT [9,10,12] verification cannot be performed using Kafka [20]. On the other hand, all other consensus algorithms bypass methods for solving the Byzantine Generals Problem, and are classified into authentication-detectable Byzantine tolerance and Byzantine tolerance.

This study presents a comprehensive taxonomy and classification of consensus algorithms, ranging from the earliest to the latest research trends. The proposed taxonomy is based on the five taxonomic ranks, including the evolutionary process and decision-making method. The study’s major contributions are identified among the five taxonomic ranks, with particular emphasis on the Order rank, where global decision-making methods are compared. For the future application of blockchain consensus algorithms, this research provides a direction, and future studies will continue to classify new and preceding consensus algorithms based on the proposed taxonomy. The aim is to classify all consensus algorithms based on reasonable criteria and manage them in the archive data format. Furthermore, a new consensus algorithm could be derived. Finally, the study concludes that consensus algorithms are crucial components of blockchain technology, enabling secure and transparent transactions by providing a way for a network of participants to reach agreement on the state of a shared database. With the increasingly anonymized cyber environment, blockchain technology is expected to be applied in various forms to industry system designs that withstand attacks from Byzantine nodes.

#### 3.3.1. Authentication-Detectable BFT Fault rank

The concept of a centralized Decision rank in the authentication-detectable Byzantine fault tolerance Fault rank does not imply that the administrator possesses absolute control, as it is centralized akin to the prevailing server–client paradigm. The degree of centralization of a consensus algorithm is determined by the existence of an entity that manages it once it has been implemented and is operational on the mainnet. In contrast, if the consensus algorithm forks every time by voting for all decisions, then it is classified as decentralized. Among the authentication-detectable Byzantine fault tolerances, the most centralized Decision rank is Feudalism, followed by Despotism, Democracy, and Liquid. Feudalism consensus algorithms include DPoS [19], Raft [27,28,121], proof of elapsed time (PoET) [29], and BFT-SMaRt [30]. DPoS [19] is a methodology in which the cryptocurrency holders exercise their voting rights in proportion to their stakes to elect their representatives and reach decisions through consensus among them. The agreement reached by nodes with stakes as representatives is similar to Feudalism, which makes decisions by forming a council. EOS [50], Lisk [51], aelf [52], Ark [53], and Bitshares [31] adopt the DPoS [19] method. The Raft [27,28] consensus algorithm was first introduced in 2014 by Diego Ongaro and John Ousterhout in a paper titled In Search of an Understandable Consensus Algorithm. It is a consensus algorithm intended to maintain all nodes in the same state in a distributed system environment and to ensure that the entire system functions without any issues even if certain nodes fail. Based on PBFT [90,91], the Raft cluster’s server is either a leader or a follower, making it centralized. The algorithm is classified as Feudalism since it is a candidate during leader elections. Another representative algorithm is the PoET [29] consensus algorithm suggested in Hyperledger Sawtooth [128]. PoET [32] is an efficient and scalable algorithm for permissioned networks. Instead of having participants solve encrypted puzzles, the algorithm generates a random model for selecting block producers, as in the resource-intensive computing in PoW [1,2] systems or PoS [18] and proof of importance (PoI) [129]. The algorithm employs required energy consumption (TEE), such as Software Guard Extension (SGX), to ensure that blocks are randomly won without necessitating any effort. A leader for generating blocks based on SGX is also selected, and as many nodes as possible participate in the consensus to fairly choose a leader, and secure CPU commands are employed to ensure safety and randomness. As a result, it is centralized and readily classified as Feudalism. Additionally, BFT-SMaRt [30] is designed to enable DoS attacks in addition to Byzantine faults. BFT-SMaRt [30] is similar to Feudalism in that fewer than one-third of all replicas of a service are faulty at any moment. The Ripple Protocol consensus algorithm [31] used by Ripple represents the Depostism Order rank and is applied every few seconds by all nodes to maintain network correctness and agreement. Once consensus is reached, the current ledger is considered closed and becomes the last-closed ledger. Ripple is owned and run by a company of the same name. Klaytn’s [54] Governance Council is an example of a democratic consensus algorithm, in which multinational corporations and organizations collaborate to manage platform governance, consensus node operations, and ecosystem expansion. This Council is comprised of high-profile global enterprises that have delivered popular user services across various domains, such as IT, telecommunications, content, gaming, and finance. Their algorithm enforces consensus between the Council’s member nodes every second, which makes it highly centralized. Masternode proof of stake (MPoS) is a consensus algorithm that integrates Masternode and PoS [18], and replaces 21 super nodes of EOS with a master node, which is eventually centralized. Significant nodes engage in proposing, voting, and community building, and MPoS approves or disapproves block creation, with additional votes being recorded in the block header. The MPoS consensus algorithm is adopted by Ether Zero [55]. On the other hand, Tezos uses the Liquid proof of stake (LPoS) [32] algorithm, categorized as the Liquid Order rank, in which a representative is randomly selected among cryptocurrency owners to make decisions through the agreement of these representatives. Hence, it is classified as a Liquid decision-making process.

The proof of stake (PoS) [18] method provides decision-making authority in proportion to the amount of stake held in a cryptocurrency, making it a Plutocracy Order rank of the de-centralized Decision rank of the authentication-detectable BFT Fault rank. Cryptocurrencies such as Ethereum [56,57], QTUM [58], Peercoin [59], and Stratis [60] use the PoS [18] method. Nodes with more coins have more opportunities to participate in block creation and receive proportional rewards. The burn and earn delegated proof of stake (B&E DPoS) consensus algorithm applied to EOS Chrome [61] allows users to receive new cryptocurrency as a reward by burning their existing coins. As a result, it is classified as a plutocratic decision-making process, where the right to verify transactions is granted in exchange for the consumption of capital. Proof of trading (PoT) returns a portion of exchange fee revenue to traders in proportion to the amount traded, and proof of burn (PoB), used in slimcoin [62], increases the likelihood of mining success in proportion to the number of coins burned. These algorithms are also considered plutocracies because nodes that participate in consensus are elected based on their capital. Tendermint [16,17] in Cosmos is almost similar to an Oligarchy, where power is concentrated among a small group of influential members. Tendermint [16,17] is created by combining DPoS [19] with practical Byzantine fault tolerance, allowing it to be used in both public and private blockchains. Tendermint [16,17] applies a locking mechanism to freeze the stakes participating in voting and prevents double voting through an unlocking mechanism, solving the nothing of stake problem. Proof of authority [21], used in Luniverse [63], replaces block miners selected based on stake in a cryptocurrency token with a small group of transaction validators selected based on their staked identity or reputation on the network. There are several published and documented algorithms that derive from PBFT [90,91], including Istanbul Byzantine fault tolerance (IBFT) [33], redundant Byzantine fault tolerance (RBFT) [34,35], and asynchronous Byzantine fault tolerance (aBFT) [36,37], which is applied to Hedera [36,37,64,65,66,67,68,69]. IBFT essentially follows the structure of PBFT [90,91] and incorporates the idea of gathering and transmitting blocks, while RBFT minimizes the performance degradation that results from a malicious primary. aBFT guarantees that honest nodes in the network agree on the timing and sequence of a set of transactions in a fair and secure manner. Ouroboros proof of stake (OPoS) [24], developed by Charles Hoskinson, is a consensus algorithm that uses a coin-tossing protocol to safeguard against ground attacks, which is a problem with existing PoS [18] methods. OPoS is employed by Cardano [130] and is a work intended for a consortium-type Cardano that seeks to solve the BFT problem by operating a small number of verification nodes without centralization. The proof of brain (PoB) [38] method is a consensus algorithm that rewards users who generate content and service participants through intellectual activity, rather than relying on miners or equity holders. This method is classified as a form of Republicanism, which involves decision making through community opinions. PoB is used by Steem, the native token of social network service (SNS) Steemit [70]. In the Spectre consensus algorithm, which is used in Spectrecoin [39], users who conduct anonymous transactions receive rewards. When a transaction is requested, cloakers randomly mix several participants to prevent traceability, and the user is rewarded accordingly. Proof of believability (PoB) is a consensus algorithm used by IOST [71] that identifies a highly reliable group based on their community contributions and groups them for verification. Proof of flow (PoF) is a consensus algorithm that rewards users who bring more traffic to the platform. In any media platform, traffic is directly linked to the platform’s value. The YOYOW [131] platform evaluates the value of its users based on their ability to bring in traffic effectively. The Algocracy of the de-centralized Decision rank of the authentication-detectable BFT Fault rank is a method of decision making based on a series of algorithms. In this study, five consensus algorithms were clustered in this category, including dual delegated proof of stake (DDPoS) [40], which uses the existing DPoS [97] method to prevent collusion between representatives by adding and verifying a sigma node that is randomly replaced according to the algorithm. Artificial intelligence delegated proof of stake (AI DPoS) is a consensus algorithm used by Velas [72], a cryptocurrency for AI-based blockchain platforms. AI DPoS autonomously selects representatives to grant block production authority using artificial intelligence (AI) functions while essentially following the existing DPoS [19] method. Proof of formulation (PoF), which selects miners based on the order of formulation rewards, was created by Fleta [73] through a partial modification of the existing delegated proof of stake. Proof of performance (PoP) is a consensus algorithm adopted by the High-Performance Blockchain (HPB) [74] that is designed to ensure both decentralization and network performance. Several variables are taken into account in the calculation, based on the performance contribution of each node. Proof of storage (PoS), used in Chia [75], is a consensus algorithm that works by having a prover store data in the free space of their hard disk, which is then approved or rejected by a verifier. It is also referred to as proof of space.

#### 3.3.2. BFT Fault Rank

The decentralized BFT Fault rank has been organized into three categories: Socialism, Anocracy, and Demarchy, which may have an atypical structure. The Socialism type is a system where workers democratically own the means of production and an autonomous economy is self-managed in a distributed environment, much like in the liberal system. In this type, the PoW algorithm [1,2] proves participation in the work by repeatedly finding a hash value that is less than the target value. The PoW [1,2] consensus algorithm implements this through mining, where miners solve a complex formula with a computer to find a hash value that meets the conditions. All nodes go through the process of verifying and approving the found hash value before storing the transaction details in the block. This algorithm has the advantage that transaction details are difficult to falsify because they must be approved by all nodes. Thus, PoW [1,2] is the consensus method that best captures the essence of decentralization on the blockchain. Satoshi Nakamoto used PoW [1,2] to mathematically ensure that all participants had the most up-to-date ledger. The Byzantine fault tolerance system has been considered a consensus algorithm in the blockchain field. PoW [1,2] was first introduced by Bitcoin [1] as a consensus structure to solve the Byzantine Generals Problem and is typically used in Bitcoin [1], Bitcoin Cash, Doge, and Litecoin. After all 21 million Bitcoins have been mined, no mining rewards are given, and Bitcoin miners only receive transaction fees. Then, it is intended to switch to a PoW [1,2] consensus algorithm. As proof of activity, miners use traditional PoW [1,2] methods to start mining by solving computational puzzles. However, only the header and the reward address of the miner, not the transactions, are included in the block mined this way. At this point, it becomes a proof of stake system, where a random group of validators is chosen to sign a new block based on the information in the header. The more stakes a validator has in a coin, the more likely they are to be selected. After verification, it becomes a full-fledged block. If some of the selected validators are unable to produce a block, the next winning block is selected, and a new validator group is created and continues until the correct number of signatures is obtained. The new validator fee is split between miners and validators accordingly. The KawPoW [118,121] algorithm is a modified version of the programmatic proof of work (ProgPoW) [41] algorithm developed by Ethereum developers to improve the ASIC resistance of Ethash [22], the EthereumPoW [76] algorithm. The Cuckoo Cycle [26,42] is the world’s first PoW algorithm developed by Dutch computer scientist John Tromp [26,42]. It uses a PoW method that uses the Cortex cuckoo cycle algorithm. The Cuckoo Cycle [26,42] is also a GPU-oriented anti-ASIC algorithm, and the difficulty and cost of mining through ASIC are higher than that of Bitcoin’s SHA-256 method. In the end, it is a slightly enhanced consensus algorithm from PoW. Dual proof of work (DPoW) is a method of mixing primary mining, which allows ASIC miners, and secondary mining, which does not allow ASIC miners. GRIN coin [71] uses this approach. Proof of useful work (PoUW) [40,72,73,131] is a consensus algorithm that uses practical computing demands to generate a new block, rather than solving a hash algorithm. This provides both energy efficiency and security and has been created by ANKR. ProgPoW [38] is an ASIC-resistant, GPU-based mining algorithm. ProgPoW [38] is a GPU-based mining algorithm and a new blockchain consensus algorithm created to disable existing application-specific integrated circuit (ASIC)-based mining. It is also a new PoW algorithm that blocks ASIC-based mining built for cryptocurrency mining on EthereumPoW [76]. It is still a complete fault tolerance structure in which all participating nodes fully utilize their resources, receive compensation for the amount they use, and voluntarily verify Byzantine faults in the network. Moving on to the Anocracy Type consensus algorithms, equilibrium proof of work (EPoW) [48] is based on the existing proof of work method, but with a unique twist. Nodes that successfully mine a block are forced to take a break for a certain period of time to distribute mining opportunities fairly among other nodes. This consensus algorithm is used by HDAC [77] Coin and enables equal opportunity and energy savings for anyone who wishes to participate in fair PoW. Spectre [39], which stands for serialization of proof of work events, is another type of consensus algorithm that is used to maintain the consensus of Hycon. It is characterized by a loosely defined form of government that is part Democracy. Silvio Micali’s PPoS [116] protocol, which is classified as a Demarchy type, is built on the basis of Byzantine consensus. The influence that each token holder has depends on the stake in the staked tokens held in the system, but users are randomly selected regardless of their stake. The fact that all online users are selected by the system increases the level of decentralization in decentralized networks and ensures system safety. This concept follows the decision-making method in Demarchy, where jurors are randomly selected from among ordinary citizens and those elected must participate in the jury.

## 4. Discussion

Overall, we provided a valuable contribution to the field of blockchain by introducing a new taxonomy and classification method for consensus algorithms, based on an evolutionary phylogeny approach. This study has introduced a new comprehensive classification taxonomy for blockchain consensuses, which is expected to aid researchers in studying various mechanisms of blockchain and its history. The taxonomy includes five main ranks, which are assigned based on subjective dissimilarity, but do not fully reflect the gradational nature of variation within the nature of blockchain, as there are no rules for how many systems of mainnet should make up a Type rank, Order rank, or other parent taxon. Nevertheless, this study represents the first complete classification of blockchain consensus algorithms, and all documented consensus algorithms have been classified in accordance with this taxonomy.

While some consensus algorithms were not included in this study due to limitations in the contents of the paper, these are not omitted but we rather focused on well-known developments in the past 20 years. The purpose of this classification of consensus algorithms is to help researchers understand the similarities and differences between various consensus algorithms, so that they can better understand current research and identify open challenges for future research, rather than duplicating research proposal ideas. When designing an efficient and ideal consensus algorithm, scalability and security are two crucial factors that must be considered. Scalability refers to the ability of a blockchain network to handle an increasing number of transactions, while security refers to the ability of the network to prevent attacks and unauthorized access. Achieving a balance between scalability and security is a challenging task, and requires careful consideration of the trade-offs between the two. Extensive research has been conducted in the blockchain field to address this issue, and several consensus algorithms have been proposed. Some of these consensus algorithms, such as the one presented in our previous research [132], aim to achieve both scalability and security, while others prioritize one over the other.

An efficient and ideal consensus algorithm in the blockchain field requires careful consideration of the trade-offs between scalability and security. While several consensus algorithms aim to achieve both, others prioritize one over the other. Thus, research on the trade-off between scalability and security is essential for the continued development and adoption of blockchain technology in various industries. Crash fault tolerance can be used under certain circumstances, such as a queue that processes only messages on a closed internal network. For isolated systems and consensus nodes that trust each other, only the consensus process needs to be undergone. Authentication-detectable BFT might be appropriate for intranetworking. The Byzantine General Problem can be easily solved under the precondition that the node’s identity is verified as friendly. A private blockchain restricts participants in advance and approves transactions only between trusted participants. Although the proof of work algorithm is the most secure and reliable among existing algorithms, it has scalability issues. Proof-of-work-based blockchains, including Bitcoin, exhibit limited transaction rates per second due to scalability issues, requiring verification and agreement of most network nodes for a new transaction block to be approved. The decentralized aspect of Bitcoin provides a secure and reliable economic system, but has inherent limitations to its use on a larger private scale, such as public data protection, logistics and distribution, electronic contract management, and food origin tracking. BFT is a method of implementing complete decentralization, and it is significant that everyone participates as a verification node and reaches a decision equally. BFT is applied to different industries, and the current belief is that blockchain can solve the Byzantine Generals Problem.

Our taxonomy, which is grounded in an evolutionary phylogeny approach, offers a comprehensive and scientific way to organize and analyze blockchain consensus algorithms. This classification method provides researchers and practitioners with a better understanding of the evolution and current state of blockchain consensus algorithms and offers potential directions for future research and development. The present study has conducted a comprehensive examination of the distinction between consensus algorithms and the relevant industry domains. Countless service sectors are emerging, including electronic cash systems, overseas remittances, over-the-counter transactions, data storage and protection, and message protection and delivery. These developments are not mere replicas, but rather stem from the reasons behind blockchain’s emergence. Specifically, it is an ideal solution to the Byzantine Generals Problem, a challenge that has persisted for over four decades. Moreover, blockchain excels in near real-time information sharing in the Internet environment, as it is free from the Byzantine Generals Problem and vice versa. As a result, there has been a long-standing desire for blockchain to flourish and its techniques to be widely adopted in industry. To achieve this goal, it is imperative to select the industry group to which blockchain technology should be initially applied. If so, it is a priority to classify numerous consensus algorithms, and then carry out a thorough review of them to predict domain-specific blockchain consensus algorithms.

## 5. Conclusions

The primary objective of this study was to provide an all-encompassing classification of consensus algorithms, tracing their evolution from the past to current trends in order to systematize the era of rapid consensus algorithm development. The research established a compendium of verified and diverse consensus algorithms, recommending popular and well-known ones, as well as grouping them into 38 clusters by identifying similarities. To investigate their correlation, the study proposed five taxonomic ranks, which included both the evolutionary process and decision-making method. This method yielded a taxonomy that can guide the future application of blockchain consensus algorithms for various industries. Our findings contributed significantly to the five taxonomic ranks, especially the Order rank, which compared global decision-making methods. Moreover, the study showed that, among the consensus algorithms in blockchains, none diverged from human decision-making methods.

As future research continues to classify the latest and preceding consensus algorithms according to our proposed taxonomy, the goal is to classify all consensus algorithms based on reasonable criteria and manage them in an archive data format. It is also conceivable that a new consensus algorithm could emerge from such research. We also hypothesize that blockchain technology will be a direct solution for solving Byzantine fault tolerance. Consensus algorithms are critical components of blockchain technology as they allow a network of participants to agree on the state of a shared database, thus ensuring secure and transparent transactions. By making sure that all participants have access to the same database version and that new transactions are authenticated and stored securely and consistently, consensus algorithms enable blockchain to function as a trusted, decentralized platform for exchanging digital assets and information. Popular examples of consensus algorithms in blockchain include proof of work, proof of stake, and delegated proof of stake. In light of the ever-growing anonymity of the cyber environment, it is anticipated that blockchain technology will be applied in various forms to industrial system designs that are capable of withstanding Byzantine node attacks.

## Figures and Tables

**Table 1:** Comparison of Byzantine fault tolerance before and after.

Classification	Description	Fault
Message Omission	Messages may be delivered, delayed, or remain undelivered.	Fail-Stop
		★ Crash
		Omission
		Performance
Message Alteration	The normality of incoming messages is uncertain.	★ Authentication-detectable Byzantine
		★ Byzantine

★: Faults level covered in this study.

**Table 2:** Comparing authentication-detectable BFT and BFT consensus algorithms.

	Authentication-Detectable BFT	BFT
Blockchain Type	Permissioned	★ Permissionless
Transaction Finality	Immediate	Immediate
Transaction Rate	High	High
Trust Model	Semi-trusted	★ Untrusted
Adversary Tolerance	<=33% [9,90,91]	

★: Noticeable difference.

**Table 3:** Synopsis of blockchain taxonomies in the decade following 2009.

No.	Survey Title	References	Year	Content
1	A taxonomy of blockchain-based systems for architecture design	Xu, Xiwei et al. [105]	2017	The present work delineates the primary structural attributes of blockchain technology and evaluates the consequences of its key design choices.
2	Taxonomy of blockchain technologies. Principles of identification and classification	Tasca, Paolo, and Claudio J. Tessone. [106]	2017	The investigation culminates in a hierarchical classification system that condenses the findings and offers a navigational instrument for traversing diverse blockchain structural configurations.
3	Analytical tools for blockchain: Review, taxonomy, and open challenges	Balaskas, Anastasios, and Virginia NL Franqueira. [107]	2018	This paper explores the existing landscape of blockchain analytic instruments and introduces a thematic taxonomic schema grounded in their functional domains. Furthermore, the study scrutinizes forthcoming research prospects and identifies persistent obstacles in the field.
4	Blockchain challenges and opportunities: A survey	Zheng, Zibin et al. [108]	2018	This publication presents a comprehensive blockchain classification system, delineates the prevalent blockchain consensus algorithms, scrutinizes an array of blockchain use cases, and examines the technical quandaries confronting the field, as well as the most recent progress in addressing these challenges.
5	A survey of blockchain from the perspectives of applications, challenges, and opportunities	Monrat, Ahmed Afif, Olov Schelén, and Karl Andersson. [109]	2019	This study delivers a comparative assessment of the cost–benefit tradeoffs of blockchain technology, clarifies the blockchain taxonomy and architecture, conducts a juxtaposition of diverse consensus mechanisms, and explores the challenges confronting the field.
6	A taxonomy of blockchain consensus methods	Nijsse, Jeff, and Alan Litchfield. [110]	2020	The paper conducts an extensive review of 19 approaches categorized by the scarce resource they employ, which include clock-cycles, bits, tokens, votes, time, and biometrics.
7	A taxonomy of blockchain threats and vulnerabilities	Alkhalifah, Ayman et al. [111]	2020	The study categorizes these occurrences according to the critical cybersecurity vulnerabilities prevalent in blockchain technologies and has devised a classification system that encompasses five different varieties of cybersecurity hazards and weaknesses, based on the roles of five primary actors within the blockchain ecosystem.
8	Verification of smart contracts: A survey	Almakhour, Mouhamad et al. [112]	2020	This paper furnishes a comprehensive summary of various methodologies for verifying smart contracts and expounds upon the tools and techniques employed. Additionally, the study appraises the strengths and weaknesses of each approach and draws definitive inferences regarding their efficacy.
9	A taxonomy of blockchain consensus protocols: A survey and classification framework	Bouraga, Sarah. [113]	2021	This research evinces that a slew of consensus proposals have emerged in a condensed timeframe and accentuates the distinctions between these protocols. The paper further posits that any forthcoming consensus protocols advanced by researchers and practitioners should take into account all dimensions proffered in the classification framework.
10	A survey and taxonomy of consensus protocols for blockchains	Singh, Arshdeep et al. [114]	2022	The paper conducts an extensive survey of consensus protocols with the express objective of identifying and expounding upon the sundry consensus protocols documented in the extant literature. In particular, the study shines a spotlight on the lineage of consensus protocols for proof-of-X, Byzantine fault tolerance, Paxos, and RAFT.
11	Formal Methods for the Verification of Smart Contracts: A Re-view	Krichen, Moez, Mariam Lahami, and Qasem Abu Al–Haija. [115]	2022	The study scrutinizes the most recent formal techniques implemented for the purpose of verifying and validating smart contract specifications with the express aim of curtailing the potential for introducing defects and bugs, as well as avoiding the attendant costs thereof.
12	Research on Progress of Blockchain Consensus Algorithm: A Review on Recent Progress of Blockchain Consensus Algorithms	Xiong, H., Chen, M., Wu, C., Zhao, Y., and Yi, W. [116]	2022	The paper introduces the fundamental concepts of blockchain technology, provides a concise overview of the principal blockchain technologies, with a particular emphasis on researching blockchain consensus algorithms, expounds upon the general principles of the consensus process, and classifies the leading consensus algorithms.
13	Blockchain for IoT Applications: Taxonomy, Platforms, Recent Advances, Challenges and Future Research Directions	Abdelmaboud, A., Ahmed, A.I.A., Abaker, M., Eisa, T.A.E., Albasheer, H., Ghorashi, S.A., and Karim, F.K. [117]	2022	This paper offers an overview and tutorial on the utilization of blockchain technology in Internet of Things (IoT) systems.
14	Blockchain-Based Internet of Things: Review, Current Trends, Applications, and Future Challenges	Alam, Tanweer [118]	2022	The paper examines the fundamental tenets of blockchain technology in the context of Internet of Things (IoT) systems, including consensus mechanisms, evaluations, challenges, opportunities, use-cases, trends, and inter-node communication within an integrated framework.

**Table 4:** Comparing prior taxonomic research with the proposed complete taxonomic classification.

Methods	Features Identified	Clustering	Distributed Field-Related	Identifying Challenges	Categorizing Domain	Lineage	Prospects
Group 1 [105,106,108,110,113]	◐	◐	✕	◐	✕	✕	✕
Group 2 [107,109,110,112,114,115,116,117,118]	✕	✕	✕	✕	⬤	✕	✕
Our classification	⬤	⬤	⬤	⬤	⬤	⬤	⬤

✕: None. ◐: On some level. ⬤: Possible.

**Table 5:** Well-known consensus algorithms.

Year	Consensus Algorithm	Type	Publication Location	Number of Citations
1982	BFT	ⓞ	★ Concurrency: the Works of Leslie Lamport [119]	8738
1989	Paxos	ⓞ	★ Concurrency: the Works of Leslie Lamport [119]	13,440
1998	Bit gold	ⓞ	Recuperado de https://nakamotoinstitute.org/bit-gold/TVer página [120]	★ 159
1999	PBFT	ⓒ	★ OsDI [90]	4927
2009	PoW	ⓞ	Bitcoin.–URL: https://bitcoin.org/bitcoin [1]	★ 25,300+
2011	Kafka	ⓒ	★ Proceedings of the NetDB [104]	1324
2011	PoS	ⓞ	Self-published paper [18]	★ 1138+
2014	DPoS	ⓢ	Bitshare whitepaper [19]	★ 328+
2014	Raft	ⓒ	★ Usenix ATC [121]	2710
2015	PoA	ⓢ	https://github.com/ethereum/guide/blob/master/poa.md [21]	unable to count
2015	Ethash	ⓢ	https://github.com/ethereum/wiki/wiki/Ethash [22]	unable to count
2016	Tendermint	ⓞ	Doctoral dissertation, University of Guelph [16]	★ 450
2017	PPoS	ⓒ	★ Proceedings of the 26th symposium on operating systems principles [23]	1345
2017	Ouroboros Byzantine	ⓒ	★ Annual international cryptology conference [24]	1590
2018	KawPoW	ⓞ	https://ravencoin.org/assets/documents/Ravencoin.pdf [25]	unable to count

ⓒ: Proceedings. ⓢ: Opensource. ⓞ: Other. ★: Featured Reference.

**Table 6:** A phylogeny approach: Defining the hierarchical taxonomy of consensus algorithms.

Main Taxonomic Ranks	Definition
Fault	Categorized according to the failure models.
Decision	Categorized according to the level of decentralization.
Order	Categorized according to the various modes of decision-making processes.
Type	Categorized according to the consensus algorithms.
System	Categorizing the mainnets.

**Table 7:** Comprehensive categorization of consensus algorithms based on taxonomic ranks.

Fault	Decision	Order	Type	System
Crash Fault Tolerance	Centralized	-	Kafka [20]	-
Authentication-detectable Byzantine Fault Tolerance	Centralized	Feudalism	Delegated Proof of Stake [19]	EOS [50]
				Lisk [51]
				aelf [52]
				Ark [53]
				BitShares [18]
			Raft [27,28]	-
			Proof of Elapsed Time [29]	Hyperledger Sawtooth [30]
			BFT-SMaRt [30]	-
		Despotism	Ripple Protocol Consensus Algorithm [31]	Ripple [31]
		Democracy	Governance Council	Klaytn [54]
			Masternode Proof of Stake	Ether Zero [55]
		Liquid	Liquid Proof of Stake [32]	Tezos [32]
	De-Centralized	Plutocracy	Proof of Stake [18]	Ethereum [56,57]
				QTUM [58]
				Peercoin [59]
				Stratis [60]
			Burn and Earn Delegated Proof of Stake	EOS Chrome [61]
			Proof of Trading	F coin
			Proof of Burn	Slimecoin [62]
		Oligarchy	Tendermint [16,17]	Cosmos
			Proof of Authority [21]	Luniverse [63]
			Istanbul Byzantine Fault Tolerance [33]	-
			Asynchronous Byzantine Fault Tolerance [36,37]	Hedera [36,37,64,65,66,67,68,69]
			Redundant Byzantine Fault Tolerance [34,35]	-
			Ouroboros Byzantine Fault Tolerance [24]	Cardano
		Republicanism	Proof of Brain [38]	Steemit [38,70]
			Proof of Anonymous Stake	Spectre [39]
			Proof of Believability	IOST [71]
			Proof of Flow	-
		Algocracy	Dual Delegated Proof of Stake [40]	Sigmachain [40]
			Artificial Intelligence Delegated Proof of Stake	Velas [72]
			Proof of Formulation	Fleta [73]
			Proof of Performance	HPB [74]
			Proof of Storage	Chia [75]
Byzantine Fault Tolerance	De-Centralized	Socialism	Proof of Work [1,2]	Bitcoin [10]
				Bitcoin Cash
				Dogecoin
				Litecoin
			KawPoW [25]	Raven [25,49]
			Ethash [22]	EthereumPoW [76]
			Cuckoo Cycle [26,42]	Cortex
			Dual Proof of Work	Grin Coin
			Proof of Useful Work [43,44,45,46,47]	ANKR
			ProgPoW [41]	-
		Anocracy	equilibrium Proof of Work [48]	Hdac [77]
			Spectre [39]	Hycon
		Demarchy	Pure Proof of Work [23]	Algorand

## Data Availability

Not applicable.

## Appendix A. The Failure Model

**Table A1:** The Model of Six Layers of Failure.

Fault	Description	
Fail-Stop	Action	When a failure occurs (Fail), no more state change occurs (Stop).
	Capability	Assuming that the requested message always arrives and that the message that arrives is normal.
	Limitation	Since it does not assume that the message does not arrive, it is impossible to handle realistic failures in which server crashes occur.
Crash	Action	Considering crashes where servers become inoperable.
	Capability	Nodes that do not respond to requested messages are assumed to have crashed. In preparation for a failure, when there is a node that does not respond, solving the problem by running a node that replaces the node.
	Limitation	It is impossible to respond to other causes by limiting the cause of the message not arriving at a crash.
Omission	Action	Without considering a single cause of the message not arriving, it is simply regarded as an omission.
	Capability	Assuming that all messages arrive within a certain amount of time or otherwise never arrive.
	Limitation	As the time when the message actually arrives cannot be determined, it does not take into account situations where the message might never arrive or arrive very late.
Performance	Action	Considering situations where messages never arrive or arrive very late.
	Capability	Used at the network level.
	Limitation	It considers whether messages arrive or not but assumes that all messages are normal.
Authentication-detectable Byzantine	Action	Among the messages that have arrived, considering even a failure that sends an abnormal message (Byzantine failure) whether intentional or unintentional.
	Capability	Used in Internet-based distributed systems.
	Limitation	Processing only verifiable among Byzantine failures.
Byzantine	Action	Blockchain.
	Capability	Handling whether the messages arrive, whether the arrived messages are normal, how to verify all failures, and how to solve the failures.
	Limitation	None.
